# Stimulation of Biological Structures on the Nanoscale Using Interfaces with Large Built-In Spontaneous Polarizations

**DOI:** 10.3390/ma17102332

**Published:** 2024-05-14

**Authors:** Nida Zia, Michael Stroscio, Mitra Dutta

**Affiliations:** 1 Electrical and Computer Engineering Department, University of Illinois at Chicago, Chicago, IL 60607, USA; nzia3@uic.edu; 2 Electrical and Computer Engineering Department, Physics Department and Richard and Loan Hill Department of Biomedical Engineering, University of Illinois at Chicago, Chicago, IL 60607, USA; 3 Electrical and Computer Engineering Department and Physics Department, University of Illinois at Chicago, Chicago, IL 60607, USA; dutta@uic.edu

**Keywords:** Debye screening, spontaneous polarization, ion channels

## Abstract

The electric potential stimulation of biological structures in aqueous environments is well-known to be a result of the gating of voltage-gated ion channels. Such voltage-gated ion channels are ubiquitous in the membranes of a wide variety of cells and they play central roles in a wide variety of sensing mechanisms and neuronal functions in biological systems. Experimental studies of ion-channel gating are frequently conducted using path-clamp techniques by placing a cumbersome external electrode in the vicinity of the extracellular side of the ion channel. Recently, it has been demonstrated that laser-induced polarization of nanoscale quantum dots can produce voltage sufficient to gate voltage-gated ion channels. This study specifically focuses on a new method of gating voltage-gated ion channels using 2D structures made of materials exhibiting large naturally occurring spontaneous polarizations, thereby eliminating the need for an external electrode or an illuminating laser. The work presents the use of self-polarizing semiconductor flakes, namely, 2H-SiC, ZnO, and GaN, to produce electric potential that is sufficient to gate voltage-gated ion channels when existing in proximity to it.

## 1. Introduction

Voltage-gated ion channels are ubiquitous in biological systems, and it is known that they can be gated by an external potential. In this paper, it is demonstrated that the naturally occurring built-in spontaneous polarization of existing 2D nano-layers that effectively serve as platforms for cells can gate voltage-gated ion channels, thereby eliminating the need for a cumbersome external electrode or external laser beam. This study specifically focuses on the gating of voltage-gated ion channels in the membranes of cells incubated in water-based suspensions of 2D nanostructures with large internal spontaneous polarizations. By modeling the external potential created in an electrolyte, with characteristic physiological properties, bordering on such materials with a spontaneous polarization, and by taking into account Debye screening in the vicinity of such surfaces, it is possible to determine the magnitude of the electric potentials produced and their ability to induce physiological effects in specific biological systems. The findings of this assessment reveal that the electric potentials generated by these semiconductor surfaces are of sufficient magnitude to stimulate the voltage-gated ion channels in biological cells. In this paper, GaN, SiC, and ZnO 2D nano-layers—with thicknesses that have recently been realized experimentally and which are known to exhibit strong naturally occurring built-in spontaneous polarizations—are modeled by including the Debye screening of the cellular electrolytes. It is shown that these 2D nano-platforms produce voltages sufficient to gate voltage-gated ion channels.

Firstly, we considered the case of silicon carbide, which is a compound semiconductor material that is gaining increasing importance as it has higher breakdown electric field strength than silicon. It exhibits attributes such as high hardness, high temperature resistance, and exceptional chemical stability. Notably, it possesses a wide bandgap of 3.26 eV, surpassing that of silicon (1.12 eV) and gallium arsenide (1.42 eV). The exceptional chemical and thermal stability of SiC enables its utilization in cutting-edge applications. Consequently, these inherent advantages contribute to its enhanced performance when compared to silicon. Moreover, SiC, like Si, has been the focus of some efforts in light of its potential biocompatibility with cellular structures [1].

**Figure 1:**
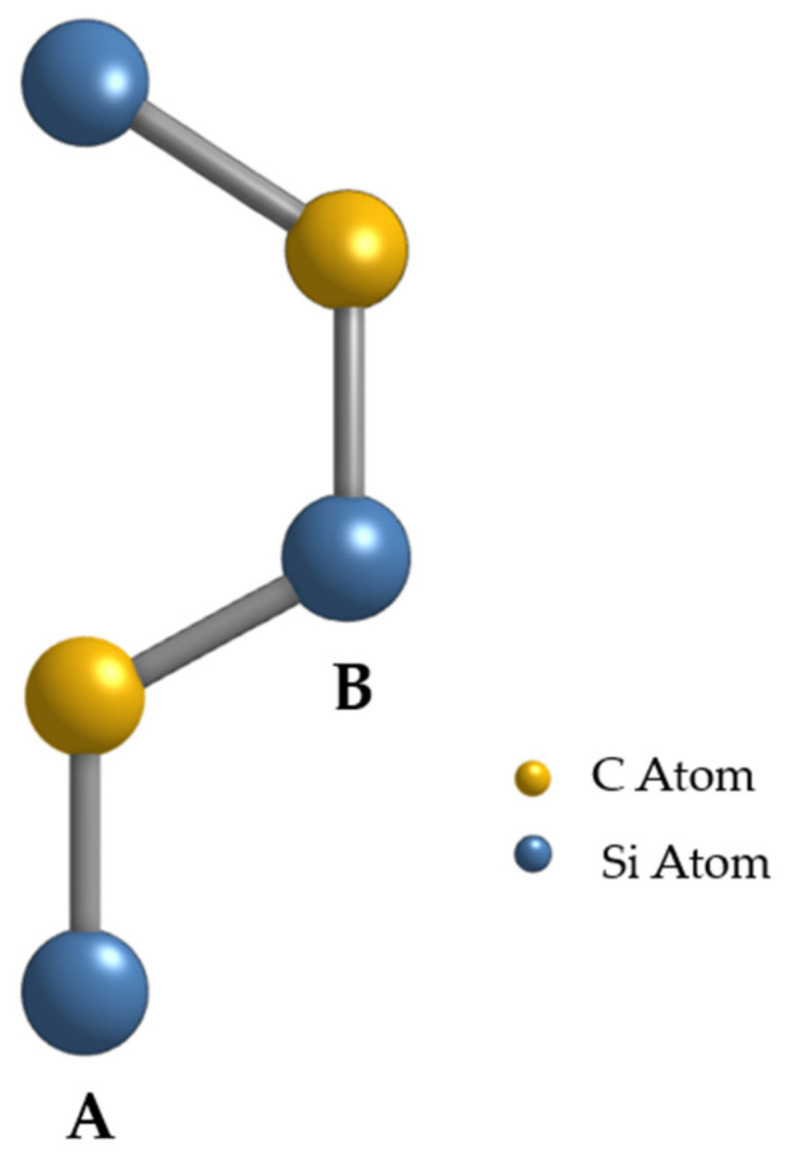
A 2H-SiCstructure that is equivalent to wurtzite and is composed of silicon (**A**) and carbon (**B**) atoms stacked as ABABAB. This illustration shows the underlying lattice structure for the hexagonal materials considered in this study; importantly, these structures are chosen because they manifest spontaneous polarizations, which are essential to the effects addressed in this paper.

In this paper, we focus on exploring the distinct physical characteristics of a polytype of SiC. Specifically, we aim to examine the electric potential generated by two-dimensional 2HSiC flakes composed of this naturally spontaneously polarized material. The hexagonal structure of this particular polytype is characterized by the …ABAB… stacking pattern as shown in Figure 1. This illustration shows the underlying lattice structure for the hexagonal materials considered in this study; importantly, these structures are chosen because they manifest spontaneous polarizations, which are essential to the effects addressed in this paper. Notably, the Monte Carlo simulation conducted on 2H-SiC has revealed that the electron mobility along the c-axis direction, which is the layer stacking direction, surpasses the electron mobility observed in both 4H- and 6H-SiC polytypes [2,3].

Due to advancements in fabricating stable 2D layers of graphene, the possibility of fabricating flakes or thin sheets of other semiconductor material is being explored. The C and Si atoms can bond through sp2 hybrid orbitals to form a SiC sheet. An attempt at fabricating ultrathin SiC nanosheets, by adopting the multistep graphene-assisted carbothermal method and post-sonication purification, is presented in [4]. It is also known that there is a slight degree of folding observed along the edges of the fabricated SiC flakes, which occurs as a means of self-stabilization upon detachment from the 3D-SiC foam. This foam is produced through carbothermal reduction involving graphene foam and SiO. The average dimensions of the 2H-SiC flakes are estimated to be around 2 μm in size, with a thickness ranging from 2 to 3 nanometers. It is also noted that the fabricated SiC flakes exhibit energetic stability.

In [5], a novel method for growing large-scale monolayer and bilayer cubic SiC crystals on a liquid Cu surface using a reproducible graphene-induced in situ process is presented. The entire growth process is carried out using the ambient pressure CVD (APCVD) method. The high growth temperature allows for the sublimation and deposition of silicon oxide (SiO_2_) obtained from a quartz tube, while the presence of liquid Cu facilitates the preformation of graphene from methane. The SiO_2_ and graphene, which are grown and reacted in situ during the CVD process, serve as the silicon and carbon sources for the cubic SiC crystals, respectively. The use of liquid Cu rather than Si wafer as a substrate enables precise control over the formation of SiO_2_ and graphene in the process. The dominant shape of the SiC flakes produced is triangular; however, rectangular, pentagonal, and even hexagonal 2H-SiC flakes are produced on the surface due to variations in the transition process. The flakes have an approximate thickness of 40 nm and a length of 21 μm.

**Figure 2:**
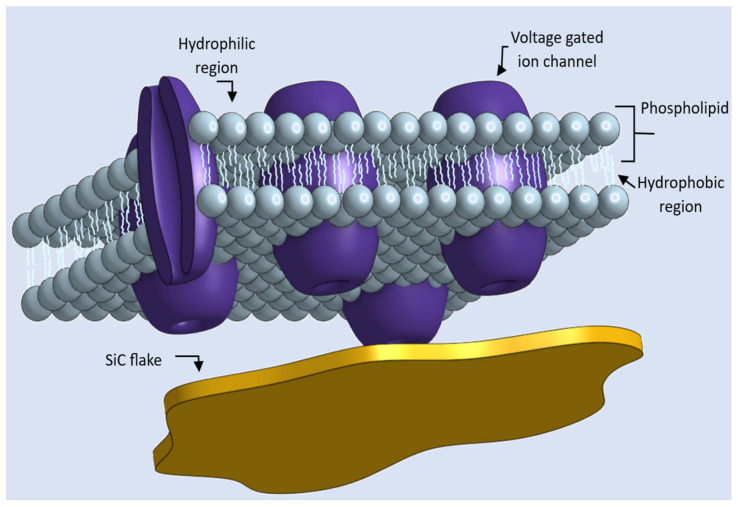
Example of an extracellular fluid comprising a self-polarizing semiconductor flake made of 2H-SiC, which is suspended around a cell. This cell is enclosed by a phospholipid bilayer membrane containing numerous channel proteins that function as ion channels.

Additionally, a technique for producing square diaphragms of 4H-SiC and 6H-SiC through a bulk micromachining process is shown in [6]. These studies define ways of fabricating hexagonal SiC surfaces. These materials include hexagonal SiC, which has been the subject of growing interest [6,7]. Moreover, there are numerous examples of the growth and fabrication of high-quality GaN and ZnO layers [8,9,10,11,12,13,14,15,16]. Importantly, sonification techniques have been demonstrated to be effective in dispersing 2D nanostructures in water [9]. Of importance for the present study, 2H-SiC, ZnO, and GaN exhibit internal built-in spontaneous polarizations that are essential for the present analysis. These self-polarizing semiconductor materials are of growing interest as their peculiar physical and chemical properties facilitate their application in various fields [17]. Now that we have established that stable SiC, ZnO, and GaN flakes can be fabricated, in this paper, we investigate the electric potential manifested by the two-dimensional surfaces made of these naturally spontaneously polarized materials in an aqueous medium that comprises cells with voltage-gated ion channels along their cell membrane, as shown in Figure 2.

Protein molecules called ion channels traverse the bilipid layer of the cell membrane, which consists of phosphate heads on both sides and fatty acid tails in between. The phosphate heads are exposed to the aqueous environment of the extracellular and intracellular fluids. The voltage-gated ion channels open and close in response to changes in the electrical properties of the membrane. The intracellular fluid is an electrolyte in the cytoplasmic interior of the cell with different ionic concentrations, while the extracellular fluid is an electrolyte with a different ionic concentration present in the extracellular region of the cell. The Nernst equation determines the inherent potential difference across the cell membrane, including the ion channels. This potential is determined by the logarithm of the ratio of ionic concentration in the extracellular region to the ionic concentration in the cytoplasmic interior of the cell. It has been observed that the intracellular region of the membrane has a significantly negative voltage compared to the extracellular region. The intracellular region typically maintains a negative potential value ranging from −0 mV to −90 mV [18]. This difference in charge, characterized by a higher concentration of anions within the intracellular region, is known as the resting membrane potential. It is well-known from patch-clamp measurements that whole-cell responses are produced when a substantial fraction of the voltage-gated ion channels is gated by an external potential [19].

When the membrane potential becomes less negative and reaches a specific threshold value associated with the channel, the membrane essentially opens, allowing selective ions to pass through. This gating process requires potentials of a few millivolts. It has been established that a potential difference of approximately 6 mV across the 7 nm thickness of an ion channel embedded in the cell membrane is sufficient for voltage-gated ion channels. Therefore, when the inherent spontaneous polarization is applied to the ion channel, originating from a 2D-SiC nanocrystal, it induces a conformational alteration. This alteration enables the diffusion of ions from the electrolyte through the ion channel, ultimately defining the open state of a voltage-gated ion channel [20].

This article models the potential in the vicinity of 2D nanocrystals made of 2H-SiC, ZnO, and GaN. It has been widely considered that the presence of spontaneous polarizations in spherical quantum dots could potentially generate a significant electric potential capable of inducing physiological effects [21,22,23,24]. The use of 2D nanostructures with built-in spontaneous polarizations, as opposed to quantum dots, presents a fundamental extension of previous studies [24]. Moreover, this model includes the effect of Debye screening, which occurs near such 2D materials when they are immersed in electrolytes that are ubiquitous in cellular environments. These results demonstrate that the potentials generated by such spontaneously polarized materials have the ability to activate the voltage-gated ion channels when a nanocrystal is in proximity. This finding implies that ion-channel gating can be achieved by employing conventional labeling methods to attach nanostructures to ion channels or by exposing cells that possess voltage-gated ion channels to a colloidal suspension of nanocrystals that possess inherent spontaneous polarizations.

This innovative approach eliminates the requirement for meticulously and precisely positioning an electrode near the ion channels of a cell, which is typically achieved in situ for cells immobilized on a substrate. An example of such an electrode is the one utilized in a patch-clamp apparatus [20] that is integrated with an optical microscope. Similarly, in [25], the voltage-gated Na+ ion channel is examined using a patch clamp and ref. [26] provides an example where a stimulating electrode is used to provide electrical stimulation of voltage-gated potassium channels.

By utilizing colloidal nanocrystals surrounded by potential fields capable of gating ion channels, the necessity for precise electrode placement near an ion channel while the cell is under observation with an optical microscope is eradicated. Moreover, it has recently been realized that the effective spontaneous polarization on the surfaces of materials with built-in spontaneous polarizations depends on the local layer-by-layer composition of selected materials manifesting spontaneous polarizations [27,28].

Motived by these effects as well as by the clear advantages of eliminating an external electrode, the present paper investigates theoretically the range of potential fields produced in aqueous environments in the vicinity of a semiconductor flake with a built-in spontaneous polarization. The present paper also analyzes the effect of Debye screening on the electric potential produced near the 2D semiconductor flakes fabricated by selected materials with spontaneous polarizations.

## 2. Materials and Methods

The potential near a surface with surface charge, σ, on a surface bounded with n electrolyte for x > 0, is,

(1) $$V\left(x\right)=\left(\frac{{\sigma \lambda }_{D}}{\epsilon_{o}\epsilon_{r}}\right)\exp(-\frac{x}{\lambda_{D}})$$

where the exponential factor accounts for the Debye screening of the potential in the electrolyte [29,30]; here, $\lambda_{D}$ is the Debye screening length, ϵo is the dielectric constant of free space, and ϵr is the relative dielectric constant of the medium; this formulation differs fundamentally from the previous paper [30], whereby the potential considered herein is for planar nanostructures while the previous paper was for spherical nanostructures. For a material with spontaneous polarization, P, σ is given by,

(2) σ=Pcosϴ

where ϴ is the angle normal to the surface of the 2D structure. For hexagonal materials, the spontaneous polarization is directed along the c-axis, which we take to be normal to the flake surface. It is known [29,30] that the Debye length in an electrolyte, given explicitly in its standard form in (3), is a function of the electrolyte concentration.

Accordingly, the Debye fall-off contributes the factor of e[−x/$\lambda_{D}$] to the potential. As Debye screening considers interactions between ions, the fall-off occurs more slowly with distance compared to the case where interactions between uncharged molecules are considered. As specified in [29,30], the Debye–Hückel approximation theory considers the case of strong electrolytes, where the accumulation of charges of the opposite sign and a deficit of charges of the same sign around an ion create an ionic atmosphere. For a monovalent ion concentration, the Debye length at room temperature is given by

(3) $$\lambda_{D}=9.62\;\mathrm{nm}/\sqrt{c}$$

where c is the ionic concentration of the electrolyte surrounding the quantum flake measured in moles per cubic meter, and 1 mole/liter equals 1000 moles per cubic meter. For reference, the typical physiological concentration of 100 moles per cubic meter, which equals 0.1 mole per liter or 100 mM, = 0.962 nm or about 1 nm. In one, two, and three Debye lengths, the Debye screening causes an additional reduction in the potential by a factor of approximately, 0.368, 0.135, and 0.050, respectively.

**Figure 3:**
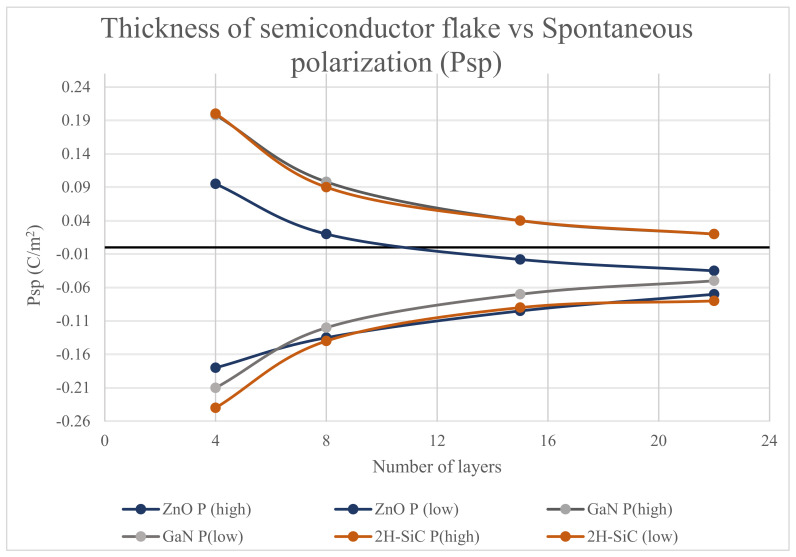
Change in the spontaneous polarization of self-polarizing semiconductor material as a function of the number of layers, or equivalently as a function of thickness of the 2D layer.

As the material with a given spontaneous polarization becomes thicker [27,28] upon stacking c1 and c2 units successively to increase the layer thickness, the values of P alternate between P (high) and P (low) forming two separate curves for P, which merge for thicknesses > 50 layers to a common asymptotic value, as shown in Figure 3. The values of the spontaneous polarizations are shown in Table 1 for ZnO, GaN, and 2H-SiC thicknesses of 4 layers, 8 layers, 15 layers, and 22 layers, respectively.

The above values are obtained using the method of [28] where the net polarizations are determined by adding the polarization of each new monolayer to the polarization of the underlying layers. The change in Psp values as a function of the number of layers for each self-polarizing semiconductor material is shown in Figure 3.

For the thinner layers, the values of P alternate above and below the asymptotic value associated bulk value, and, importantly, can be significantly larger than the bulk value of P for a given semiconductor with a built-in spontaneous polarization. Calculated and measured values of the bulk spontaneous polarization for ZnO [27] are −0.04954 C/m^2^ [28] and −0.057 C/m^2^ [31], respectively. For GaN, calculated and measured values of the bulk spontaneous polarization are −0.01975 C/m^2^ [27] and −0.018 to −0.023 C/m^2^ [32], respectively. For 2H-SiC, calculated and measured values of the bulk spontaneous polarization are −0.02861 C/m^2^ [27] and −0.0111 to −0.0432 C/m^2^ [33], respectively.

Taking the P (low) values for ZnO, GaN, and SiC for thicknesses with 4 layers, the polarizations at x = 0 are 180 mC/m^2^, 210 mC/m^2^, and 24 mC/m^2^, respectively. The surface potential for ZnO, GaN, and SiC are found to be 244 mV, 285 mV, and 33 mV, respectively; in determining these values, the relative dielectric constant of the water adjacent to the material with spontaneous polarization has been taken to be 80. These potentials are sufficient to gate a voltage-gated ion channel in the case where these potentials are dropped over the thickness of the ion channel, since potentials of 6 mV are known to be sufficient for such processes [23,26,34,35].

## 3. Results

Based on the spontaneous polarization values provided in Table 1, we can determine the electric potential generated near a 2D SiC, GaN, and ZnO nanocrystal submerged in an electrolyte. To select between P (high) and P (low) from Table 1, it is necessary to know the precise thickness of the 2D nanocrystal. However, for the calculation of the electric potential, we illustrate our results for the Psp (low) value since the many-monolayer limit for known spontaneously polarized materials saturates to a negative value. Using this value and (1), we calculate the electric potential produced in the vicinity of the semiconductor interface made of these self-polarizing materials with built-in spontaneous polarizations.

The ability of the 2D semiconductor interface to create a physiological effect is evaluated in this paper in terms of the distance from the interface to an arbitrary point, x. In this assessment, distances of 0.5 nm and 1 nm are considered. At these distances, the electric potential generated is found to be strong enough to induce a physiological response. To study the impact of Debye screening on the electric potential, the values of Debye length are varied from 0.33 nm to 2.33 nm. These specific values of the Debye length were selected as they represent the range of interest in physiological environments [36,37,38,39]. The values of the electric potential (in volts) are shown in Table 2, Table 3, Table 4, Table 5, Table 6 and Table 7 for ZnO, GaN, and 2H-SiC flakes. The semiconductor flake thickness of 4 layers, 8 layers, 15 layers, and 22 layers is converted to nanometers units (nm)—using lattice constants of 0.5206 nm, 0.5185 nm, and 0.5110 nm, for ZnO, GaN, and 2H-SiC, respectively—for respective self-polarizing semiconductor material considering their lattice constant. Each material has a different lattice constant in the c-direction, which is the relevant direction for the spontaneous polarization effect. The lattice constants of ZnO, GaN, and 2H-SiC are 0.5206 nm, 0.5185 nm, and 0.5110 nm, respectively.

**Figure 4:**
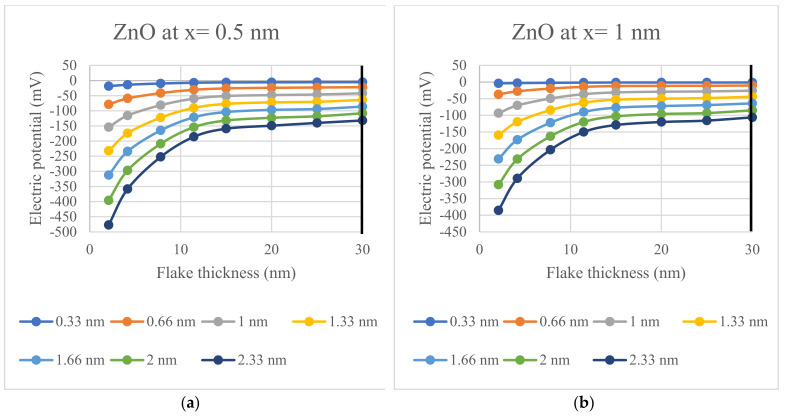
The calculated electric potential for a ZnO semiconductor interface for Debye lengths ranging between 0.33 nm and 2.33 nm: (**a**) Electric potential calculated for x = 0.5 nm; (**b**) Electric potential calculated for x = 1 nm.

**Figure 5:**
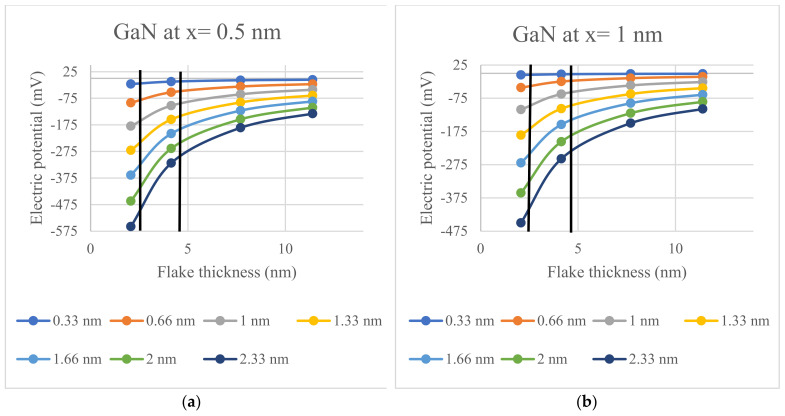
The calculated electric potential for a GaN semiconductor interface for Debye lengths ranging between 0.33 nm and 2.33 nm: (**a**) Electric potential calculated for x = 0.5 nm; (**b**) Electric potential calculated for x = 1 nm.

**Figure 6:**
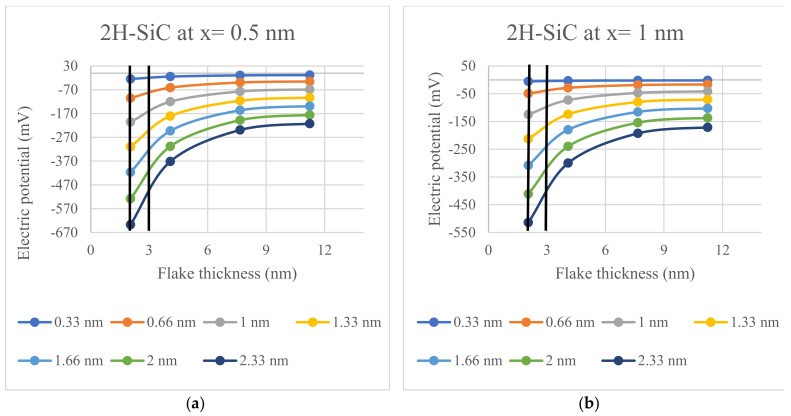
The calculated electric potential for a 2H-SiC semiconductor interface for Debye lengths ranging between 0.33 nm and 2.33 nm: (**a**) Electric potential calculated for x = 0.5 nm; (**b**) Electric potential calculated for x = 1 nm.

The electric potential trends, as a function of semiconductor flake thickness and Debye length, are illustrated in Figure 4, Figure 5 and Figure 6; all figures have been prepared with ExCel. The thick vertical lines on the plots mark the fabricable flake thickness of respective semiconductor materials.

## 4. Discussion

By modeling the potential in the vicinity of 2D nanocrystals made of 2H-SiC, ZnO, and GaN, it is demonstrated that the recently fabricated 2D nanostructures produce voltages that are sufficient to gate voltage-gated ion channels. Specifically, the electric potentials of Figure 4, Figure 5 and Figure 6 are given by the potential of (1) for the spontaneous polarization of each material and thickness.The values of the potentials in Figure 4, Figure 5 and Figure 6 are presented since they represent cases that are likely to be encountered in practical applications; however, the potential given by (1) can used for any desired layer thickness, distance from layer, and electrolyte concentration. Critically, this model includes the effect of Debye screening, which occurs near such 2D materials when they are immersed in electrolytes that are ubiquitous in cellular environments. The results of Figure 4, Figure 5 and Figure 6 demonstrate that the potentials generated by such spontaneously polarized 2D materials, which have thicknesses that have been realized for a variety of synthesis studies, are multiples of 10 mV over a wide range of physiological conditions and have the ability to activate the voltage-gated ion channels when a nanocrystal is in proximity. Accordingly, these potentials are sufficient to switch voltage-gated ion channels, which are ubiquitous in cellular membranes. These findings demonstrate the feasibility of producing physio-logical effects using 2D nanostructures fabricated of materials exhibiting built-in spontaneous polarizations. These findings are particularly meaningful in that they eliminate the need to use traditional cumbersome techniques to apply potential voltages across ion channels. We mention, parenthetically, that there are many other possible applications of 2D layers in biological applications such as those incorporating field-effect transistors [40], but such uses of 2D structures are not based on the underlying phenomena presented in the current paper.

## 5. Conclusions

These results indicate that voltage-gated ion channels may be gated by 2D nano-platforms composed of ZnO, GaN, and 2H-SiC flakes of thicknesses that have been produced in a number of fabrication efforts. This eliminates the need for an external electrode or an external laser beam since these 2D nano-platforms produce potentials that are sufficient to switch voltage-gated ion channels in cellular membranes in typical physiological electrolytes. Specifically, such gating voltages are produced over a wide range of Debye screening lengths. These results are based on the existence of substantial electric potentials along the c-axis of the wurtzite crystals in the ZnO, GaN, and 2H-SiC 2D semiconductor layers, which are found as a result of their built-in spontaneous polarizations. The modeled potentials, taking into account the Debye screening effect, are found to be substantially greater than those needed to produce physiological effects in cellular structures containing voltage-gated ion channels.

## Figures and Tables

**Table 1:** The spontaneous polarization values for ZnO, GaN, and 2H-SiC of 4-, 8-, 15-, and 22-layer thicknesses.

	ZnO P (High) C/m^2^	ZnO P (Low) C/m^2^	GaN P (High) C/m^2^	GaN P (Low) C/m^2^	2H-SiC P (High) C/m^2^	2H-SiC P (Low) C/m^2^
4 layers	0.095	−0.180	0.198	−0.210	0.20	−0.24
8 layers	0.020	−0.135	0.098	−0.120	0.09	−0.14
15 layers	−0.018	−0.095	0.040	−0.070	0.04	−0.09
22 layers	−0.035	−0.070	0.020	−0.050	0.02	−0.08

**Table 2:** Electric potential produced by ZnO flake at x = 0.5 nm.

Debye Length $\lambda_{D}$	0.33 nm	0.66 nm	1 nm	1.33 nm	1.66 nm	2 nm	2.33 nm
Flake Thickness							
2.08 nm	−0.018	−0.078	−0.154	−0.232	−0.312	−0.395	−0.477
4.16 nm	−0.013	−0.059	−0.115	−0.174	−0.234	−0.297	−0.358
7.8 nm	−9.72 × 10^−3^	−0.041	−0.081	−0.122	−0.164	−0.208	−0.252
11.45 nm	−7.16 × 10^−3^	−0.030	−0.059	−0.09	−0.121	−0.154	−0.186
15 nm	−6.14 × 10^−3^	−0.026	−0.051	−0.077	−0.104	−0.132	−0.159
20 nm	−5.73 × 10^−3^	−0.024	−0.048	−0.072	−0.097	−0.123	−0.149
25 nm	−5.53 × 10^−3^	−0.023	−0.046	−0.070	−0.094	−0.118	−0.140
30 nm	−5.07 × 10^−3^	−0.022	−0.042	−0.064	−0.086	−0.108	−0.132

**Table 3:** Electric potential produced by ZnO flake at x = 1 nm.

Debye Length $\lambda_{D}$	0.33 nm	0.66 nm	1 nm	1.33 nm	1.66 nm	2 nm	2.33 nm
Flake Thickness							
2.08 nm	−4.05 × 10^−3^	−0.037	−0.093	−0.159	−0.230	−0.308	−0.385
4.16 nm	−3.03 × 10^−3^	−0.027	−0.070	−0.119	−0.173	−0.231	−0.289
7.8 nm	−2.13 × 10^−3^	−0.019	−0.049	−0.084	−0.121	−0.162	−0.203
11.45 nm	−1.57 × 10^−3^	−0.014	−0.036	−0.061	−0.089	−0.119	−0.149
15 nm	−1.35 × 10^−3^	−0.012	−0.031	−0.053	−0.077	−0.103	−0.129
20 nm	−1.26 × 10^−3^	−0.012	−0.029	−0.050	−0.072	−0.096	−0.120
25 nm	−1.22 × 10^−3^	−0.011	−0.028	−0.048	−0.069	−0.093	−0.116
30 nm	−1.11 × 10^−3^	−0.010	−0.026	−0.044	−0.064	−0.085	−0.106

**Table 4:** Electric potential produced by GaN flake at x = 0.5 nm.

Debye Length $\lambda_{D}$	0.33 nm	0.66 nm	1 nm	1.33 nm	1.66 nm	2 nm	2.33 nm
Flake Thickness							
2.07 nm	−0.0215	−0.091	−0.179	−0.270	−0.364	−0.461	−0.557
4.14 nm	−0.0122	−0.052	−0.103	−0.155	−0.208	−0.264	−0.318
7.7 nm	−7.16 × 10^−3^	−0.030	−0.059	−0.090	−0.121	−0.154	−0.186
11.4 nm	−5.11 × 10^−3^	−0.022	−0.043	−0.064	−0.087	−0.110	−0.133

**Table 5:** Electric potential produced by GaN flake at x = 1 nm.

Debye Length $\lambda_{D}$	0.33 nm	0.66 nm	1 nm	1.33 nm	1.66 nm	2 nm	2.33 nm
Flake Thickness							
2.07 nm	−4.72 × 10^−3^	−0.043	−0.109	−0.186	−0.269	−0.360	−0.450
4.14 nm	−2.7 × 10^−3^	−0.024	−0.062	−0.106	−0.154	−0.205	−0.257
7.7 nm	−1.575 × 10^−3^	−0.014	−0.036	−0.062	−0.090	−0.119	−0.150
11.4 nm	−1.12 × 10^−3^	−0.010	−0.026	−0.044	−0.064	−0.086	−0.107

**Table 6:** Electric potential produced by 2H-SiC flake at x = 0.5 nm.

Debye Length $\lambda_{D}$	0.33 nm	0.66 nm	1 nm	1.33 nm	1.66 nm	2 nm	2.33 nm
Flake Thickness							
2.04 nm	−0.025	−0.105	−0.205	−0.309	−0.416	−0.528	−0.637
4.08 nm	−0.014	−0.061	−0.120	−0.180	−0.243	−0.308	−0.372
7.66 nm	−9.21 × 10^−3^	−0.039	−0.077	−0.116	−0.156	−0.198	−0.239
11.24 nm	−8.19 × 10^−3^	−0.035	−0.068	−0.103	−0.139	−0.176	−0.212

**Table 7:** Electric potential produced by 2H-SiC flake at x = 1 nm.

Debye Length $\lambda_{D}$	0.33 nm	0.66 nm	1 nm	1.33 nm	1.66 nm	2 nm	2.33 nm
Flake Thickness							
2.04 nm	−5.4 × 10^−3^	−0.049	−0.125	−0.212	−0.308	−0.411	−0.514
4.08 nm	−3.15 × 10^−3^	−0.029	−0.073	−0.124	−0.179	−0.240	−0.299
7.66 nm	−2.02 × 10^−3^	−0.018	−0.047	−0.079	−0.115	−0.154	−0.193
11.24 nm	−1.8 × 10^−3^	−0.016	−0.042	−0.071	−0.103	−0.137	−0.171

## Data Availability

All data used and/or analyzed during the current study are shown within the study. If further data are required, they can be made available by the corresponding author upon reasonable request.
